# Atovaquone attenuates experimental colitis by reducing neutrophil infiltration of colonic mucosa

**DOI:** 10.3389/fphar.2022.1011115

**Published:** 2022-10-14

**Authors:** Laura D. Manzanares, Joseph David, Xingsheng Ren, Lenore K. Yalom, Enzo B. Piccolo, Yalda Dehghan, Aidan J. David, Stephen B. Hanauer, Ronen Sumagin

**Affiliations:** ^1^ Laboratory 7-065 Department of Pathology, Northwestern University Feinberg School of Medicine, Chicago, IL, United States; ^2^ Department of Medicine, Gastroenterology and Hepatology University of Arizona College of Medicine, Phoenix, AZ, United States; ^3^ Department of Medicine, Northwestern University Feinberg School of Medicine, Chicago, IL, United States; ^4^ College of Arts and Sciences, Case Western Reserve Unviersity, Cleveland, OH, United States; ^5^ Department of Medicine, Gastroenterology and Hepatology Northwestern Memorial Hospital, Chicago, IL, United States

**Keywords:** atovaquone, inflammation, ulcerative colitis, drug repurposing, neutrophils

## Abstract

Ulcerative colitis (UC) is a chronic relapsing disease featuring aberrant accumulation of neutrophils in colonic mucosa and the luminal space. Although significant advances in UC therapy have been made with the development of novel biologics and small molecules targeting immune responses, success of most current therapies is still limited, with significant safety concerns. Thus, there is a need to develop additional safe and effective therapies for the treatment of UC. Antimalarial drugs have been safely used for many years to resolve tissue inflammation and the associated pathologies. Atovaquone is a recent FDA-approved antimalarial drug that has shown anti-viral and tumor-suppressive properties *in vitro* however, its role in mucosal inflammation has not been evaluated. Using pre-clinical murine DSS-induced colitis model combined with complementary *in vivo* peritonitis and *ex vivo* human neutrophil activation and chemotaxis assays we investigated functional and mechanistic impacts of atovaquone on disease resolution and neutrophil trafficking. We demonstrate that atovaquone promotes resolution of DSS-induced murine colitis by reducing neutrophil accumulation in the inflamed colonic mucosa. Mechanistically, we show that atovaquone suppressed induction of CD11b expression in neutrophils, reducing their polarization and migratory ability. Thus, our findings identify a new role of atovaquone in promoting resolution of mucosal inflammation, supporting the idea of potential repurposing of this FDA-approved drug as UC therapeutic.

## Introduction

Ulcerative colitis (UC) is a chronic relapsing disease characterized by diffuse mucosal inflammation of the colon, driven by a dysregulated inflammatory response in genetically susceptible individuals (Danese and Fiocchi, 2011). Defects in the IL-10R/IL-10 axis, aberrant activation of T cell responses leading to elevated production of pro-inflammatory cytokines and chemokines such as IL-17, IFN-γ, IL-6, IL-23, TNFα, and CXCL8, as well as neutrophil accumulation in inflamed colonic mucosa have all been associated with increased disease severity (Ordás et al., 2021).

Neutrophils are the first responders to an inflammatory insult and their primary function is host defense and elimination of extracellular pathogens *via* phagocytosis, production of reactive oxygen species, degranulation, release of extracellular traps and secretion of cytokines and chemokines that allow cellular crosstalk with other immune cells (Rosales, 2018). These mechanisms are important for the maintenance of gut homeostasis, but under persistent inflammatory conditions, neutrophil function can become deleterious. Indeed, neutrophil presence in inflamed colonic mucosa is typically viewed as pathologic and has been correlated with disease severity (Lampinen et al., 2005; Bennike et al., 2015; Therrien et al., 2019). Hence, neutrophil function and presence in the colon is of paramount importance.

Infiltration and accumulation of immune cells, particularly neutrophils, in inflamed colonic tissue and luminal spaces is a multi-step process that requires tightly regulated interactions with endothelial and epithelial barrier forming cells, as well as establishment of chemotactic gradients (Voisin and Nourshargh, 2013; Sumagin et al., 2014). Hence, it has been of continued interest to develop specific therapies targeting adhesion molecules and chemotactic factors aimed at reducing immune cell tissue recruitment (Sumagin et al., 2021). Aminosalicylates (mesalamine formulations) and corticosteroids are typically used as first-line therapy in mild to moderate UC, while immunomodulators, monoclonal antibodies, and small molecules are used for the treatment of moderate to severe disease (Danese et al., 2020). Several biologic agents have been developed and are currently in use for treatment of UC, including antagonists of anti-TNFα (infliximab and adalimumab), α4β7 integrin (vedolizumab) and IL-12/23 (ustekinumab). More recently, small molecules inhibiting janus kinase (tofacitinib, upadacitinib) and sphingosine 1 phosphate (ozanimod) have been approved for the treatment of moderate to severe UC. However, all of these drugs have potentially serious side effects and only moderate success rates with generally about one third to one half of UC patients responding to such therapies (Veny et al., 2020). Further, these drugs are expensive and generally used life-long. Given these factors, and the fact that UC is responsible for considerable morbidity with a rising incidence globally, there is a need for developing safe, effective, and less expensive therapies for the treatment of UC (Ananthakrishnan et al., 2020).

Anti-malarial drugs such as hydroxychloroquine have been safely used for decades in patients with various inflammatory conditions. With regards to mucosal inflammation, hydroxychloroquine has been shown to attenuate murine colitis and the development of colitis associated colon cancer (CAC) (Yao et al., 2016). However, a human study failed to show hydroxychloroquine superiority over placebo in 93 UC patients (Fiorillo et al., 2016). A subsequent study comparing chloroquine to sulfasalazine in 60 human subjects found equal or better rates of remission and response, and fewer adverse events in the subjects treated with chloroquine (Goenka et al., 1996).

Atovaquone is an anti-protozoal agent belonging to a group of compounds called synthetic hydroxynaphthoquinones and has been widely used in the prevention and treatment of malaria as well as other protozoal diseases. Its anti-malarial properties stem from its inhibition of the parasite mitochondrial electron transport chain by acting as a competitive inhibitor of ubiquinone which causes collapse of the mitochondrial membrane potential. Recently, atovaquone has been discovered to have tumor-suppressive and anti-viral properties against SARS-CoV-2 *in vitro* (Fiorillo et al., 2016; Carter-Timofte et al., 2021). However, its role in mucosal inflammation has never been determined. In this study, we demonstrate for the first time that atovaquone promotes resolution of DSS-induced murine colitis. Importantly, we show that the beneficial impact of atovaquone treatment is due to decreased expression of neutrophil migration regulating αM integrin (CD11b) and a robust inhibition of neutrophil infiltration of the inflamed colonic mucosa. Thus, our data indicate that atovaquone exerts anti-inflammatory/pro-resolution effects in mucosal inflammation *via* direct regulation of neutrophil activation, and by limiting neutrophil tissue accumulation.

## Materials and methods

### Mice

C57BL/6J mice (Jackson Laboratories, Bar Harbor, ME, United States) aged 8–12 weeks were maintained under specific pathogen-free conditions at Northwestern University, Feinberg School of Medicine animal facilities. All experimental procedures were approved by the Northwestern Institutional Animal Care and Use Committee.

### DSS-induced colitis model

Mice (*n* = 5 per group) were given 3% DSS (molecular mass, 40,000, Alfa Aesar, MA, United States) in drinking water for 5 days to induce colitis followed by regular water or atovaquone (10 mg/kg) for 7 days (Kiesler et al., 2015). Disease severity was assessed daily with Disease activity index (DAI) score calculated based on combined score of weight loss: (0—no loss, 1—1%–5%, 2—5%–10%, 3—10%–20% and 4—>20% loss), intestinal bleeding: 0—no blood, 2—hemoccult positive, 3—gross blood in pellet and 4—visible rectal bleeding, and stool consistency: (0—normal, 2—loose stool and 3—diarrhea). Atovaquone concentration was determined according to established dosage for human anti-malarial treatment with atovaquone/proguanil.

### Isolation of immune cells from lamina propria

Colons were dissected from healthy controls and from DSS-treated mice with and without atovaquone. For lamina propria (LP) immune cells isolation, epithelium was removed by two EDTA (Thermofisher, MA, United States) shakes (30 min at 4°C) followed by tissue digest using collagenase solution (1.5 mg/ml type VIII collagenase, Sigma-Aldrich, MO, United States) dissolved in HBSS (Corning, NY, United States)/FBS (R&D Systems, MN,United States) with 40 μg/ml of DNase I (New England BioLabs, MO, United States) at 37°C for 45 min at 150 rpm (Geem et al., 2012). Isolated immune cells were used for flow cytometry analyses as detailed below.

### Isolation of peripheral blood neutrophils

For murine neutrophils, uncoagulated blood samples (300 μl) were obtained from healthy, DSS-colitis, and zymosan induced peritonitis mice, treated or untreated with atovaquone. Red blood cells (RBC) were lysed with ACK lysis buffer (Gibco, MA, United States) and immune cells were prepped and stained for flow cytometry. Human blood was drawn from healthy donors according to approved procedures by the Northwestern University Institutional Review Board (STU00212938) and layered on top of 5 ml of polymorphprep (Thermofisher, MA, United States). Neutrophils were obtained through separation of blood cells based on density as has been previously described (Butin-Israeli et al., 2016; Butin-Israeli et al., 2019). Neutrophils were washed with an equal volume of 0.45% NaCl, and RBC were lysed with sterile ice-cold water followed by addition of 1.8% NaCl to restore osmolarity. Neutrophils were centrifuged and resuspended in appropriate volume of HBSS (Corning, NY, United States).

### Chemotaxis assay

Chemotaxis assays were performed according to a previously described protocol (Rehring et al., 2021). Briefly, transwell filters (3.0 μm pore size, CoStar Group, DC, United States) were coated overnight with 50 μg/ml collagen I (Corning, NY, United States) in 0.2% acetic acid buffer. 1 × 10^6^ human neutrophils were loaded into the upper chamber of a 24 well transwell plate (Corning, NY, United States) and induced to chemotax towards an fMLF gradient 200 nM in 500 μl HBSS+ at 37°C for 1 h. The number of neutrophils that migrated to the bottom chamber was quantified by counting 10 randomly selected fields using an inverted phase-contrast microscope (Leica Microsystems, DEU).

### Flow cytometry

Immune cells from blood, peritoneal lavage or lamina propria digestion were collected and stained for flow cytometry. Cells were incubated with fluorescently conjugated antibodies anti-mouse CD45-PB (BioLegend, CA, United States) anti-Ly6G-BV510 (BioLegend, CA, United States), anti-mouse CD11b-ApCy7 (BioLegend, CA, United States) anti-human CD66b-PE (BioLegend, CA, United States) and anti-human CD11b-FITC clone: CBRM1/5 (BioLegend, CA, United States) for 20 min at 4°C in the dark. Cells were washed with cold PBS twice, centrifuged and resuspended in appropriate volume. Cells were then analyzed by FACS LSRFortessa (BD Biosciences) and FlowJo software (Tree Star, OR, United States). Neutrophils were gated as CD11b+/Ly6G+ cells.

### Immunohistochemistry

4% PFA-fixed paraffin-embedded tissues were sectioned at 5 μm and mounted onto Apex Superior Adhesive Slides (Leica, Wetzlar, Germany). Standard deparaffinization was done in xylene and serial alcohol immersion. Heat-induced epitope retrieval was performed in eBioscience IHC Antigen Retrieval Solution, pH 6.0 (Thermo Scientific) by pressure cooking at 125°C for 30 s and gradual cooling to 90°C over 40 min. Slides were stained using Dako Autostainer Plus (Dako Omnis; Agilent, Santa Clara, CA, United States) slides were rinsed with buffer and blocked with H_2_O_2_ block for 10-min, Dako envision kit Protein Block (Dako Omnis). Slides were incubated with S100A9 primary antibody (1:3000 dilution, NB110–89726; Novus Biologicals) for 1 h with a posterior 30-min Dako Rabbit Labeled Polymer secondary antibody (Dako Omnis), washed (×2) for 7-min with Dako DAB + Chromogen (Dako Omnis), and counterstaining with blue Mayer’s Hematoxylin (Sigma Aldrich). Slides were imaged and scanned using a NanoZoomer 2.0-HT. Images were analyzed by NDP.view2 Viewing software (Hamamatsu Photonics, Hamamatsu, Japan) and ImageJ.

### Zymosan-induced peritonitis model

Zymosan injection 2 μg/ml (Sigma-Aldrich,MO, United States) in 500 μl PBS, ip. was used to induce peritoneal inflammation and neutrophil recruitment into the peritoneal cavity as previously described (Slater et al., 2017). 4 h post injection, peritoneal content was harvested through lavage with 4 ml of PBS (Corning, NY, United States) immune cells were prepped and stained for flow cytometry. Neutrophils were defined and gated as CD45^+^/CD11b^+^/Ly6G^+^. Neutrophil numbers were calculated by multiplying the number of cells collected in the lavage and the percentage of neutrophils determined by flow cytometry per 1 ml of collected lavage.

### *Ex vivo* whole mount imaging

Following zymosan treatment, mice were anesthetized and a peritoneal flap was created by a small incision of the skin and the abdomen wall to expose the mesenteric vasculature. Abdominal segment was secured to a silicone gel plate using Vetbond tissue adhesive (3 M). All imaging experiments were performed using Olympus BX-51WI spinning disk confocal microscope, equipped with a Yokogawa CSU-X1-A1 spinning disk, a Hamamatsu EMCCD C9100–50 camera, and a Modular Laser System with solid state diode lasers with DPPS modules for 488, 561, and 640 nm and the appropriate filters (all assembled by Perkin Elmer, Naperville, IL, United States). Z-axis movement and objective positioning was controlled by Piezoelectric MIPOS100 System (Piezoystem Jena, Germany). Images were collected using a 20× water-immersion objective (1.00 numerical aperture) and analyzed off-line using ImageJ software (NIH, Bethesda MD).

### High-resolution endoscopy

To maintain appropriate levels of anesthesia throughout the procedure, mice were anesthetized with ketamine (100 mg/kg) and xylazine mixture (5 mg/kg). Endoscopic imaging on anesthetized mice was performed using Storz high-resolution endoscope. For all conditions the colon was examined for inflammatory alterations such as mucus built-up, swelling and bleeding.

### Biological target prediction

Hydroxychloroquine and atovaquone chemical structures were retrieved from PubChem NCBI (https://pubchem.ncbi.nlm.nih.gov/) and uploaded to PharmMapper server (http://www.lilab-ecust.cn/pharmmapper/). The targets obtained from PharmMapper with the highest scores where then assessed through gene ontology (http://geneontology.org/) for biological process identification and to KEGG pathway analysis (https://www.genome.jp/kegg/pathway.html) for the identification of pathologies associated with the predicted targets.

### Statistics

All data are presented as mean ± SD. Statistical significance was set at *p* < 0.05 and was determined by one-way ANOVA and two-way ANOVA for matched data sets for DAI analyses using Prism, ver. 9.0 (GraphPad, La Jolla, CA, United States).

## Results

### Atovaquone is predicted to have unique biological targets and functions in disease

**FIGURE 1:**
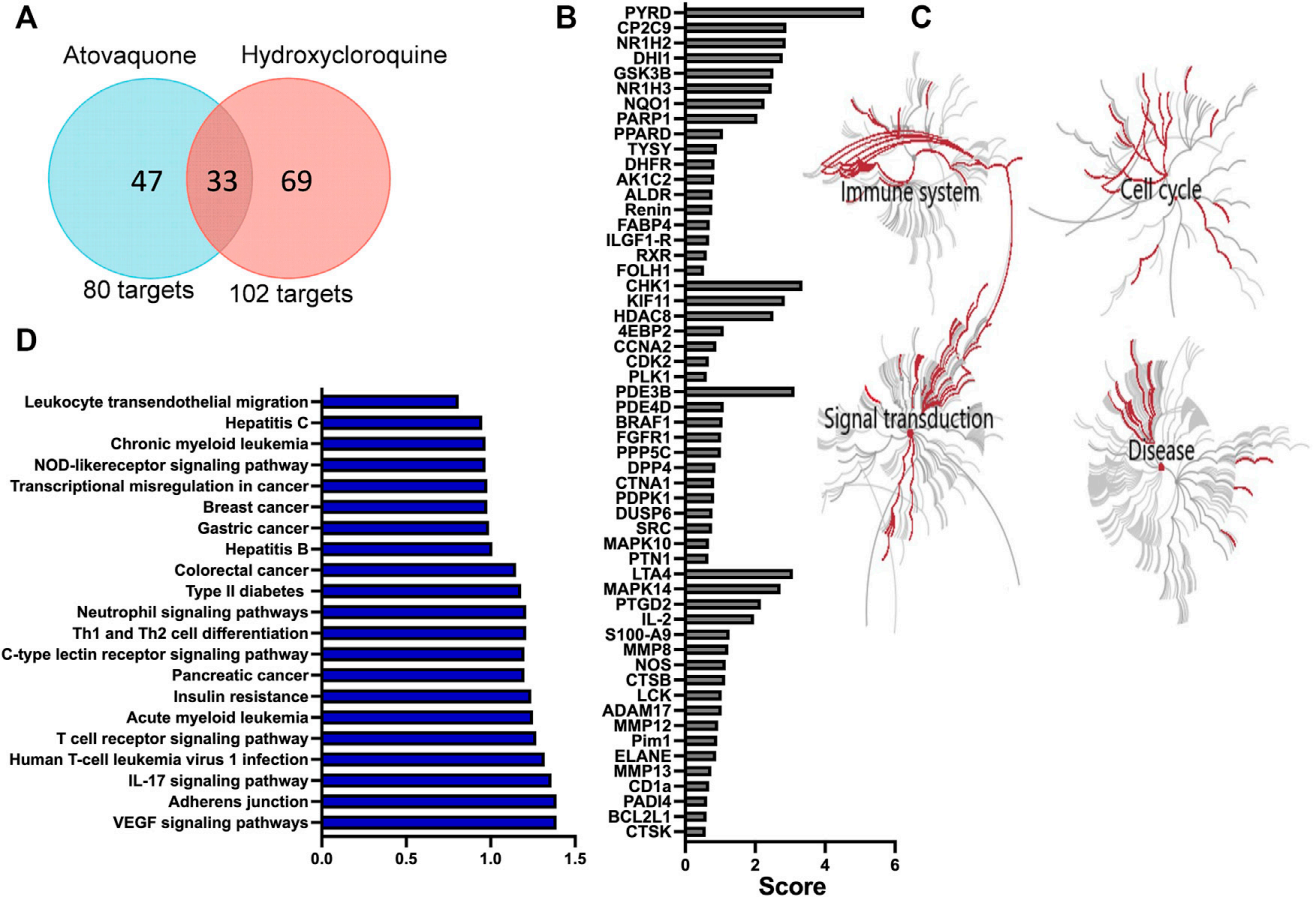
Atovaquone is predicted to have unique biological targets and functions in disease. **(A)** Comparison of atovaquone and hydroxychloroquine predicted targets using PharmMapper. **(B)** Top predicted targets of atovaquone according to PharmMapper. **(C)** GO analysis predicted biological functions targeted by atovaquone. **(D)** KEGG analysis identification of disorders associated with atovaquone targets.

Hydroxychloroquine is an anti-malarial drug with immunomodulatory properties, which has been shown to attenuate murine colitis and suppress development of CAC (Yao et al., 2016). Atovaquone is another FDA approved anti-malarial drug, which has similarly shown tumor-suppressive and anti-viral properties *in vitro* (Fiorillo et al., 2016; Carter-Timofte et al., 2021). However, its role in mucosal inflammation has not been investigated. To address this, we used a prediction algorithm [PharmMapper (Liu et al., 2010; Wang et al., 2016; Wang et al., 2017)] to identify and map potential targets of atovaquone and compared these to hydroxychloroquine. As expected, given structural similarities, we identified 33 shared targets of atovaquone and hydroxychloroquine. However, intriguingly, 47 targets were identified as specific to atovaquone (Figure 1A), indicating potentially unique functional specialization. To further identify functionalities related to suggested targets, we performed GO analysis, which revealed enrichment of four distinct clusters associated with cell cycle, signal transduction, immune system activation and disease progression. Alterations in cell cycle were expected since atovaquone targets purine and pyrimidine biosynthesis in malaria parasites through mitochondria disruption. Interestingly, immune modulation, and in particular that of neutrophils, has also been predicted to be impacted by atovaquone treatment. We found that S100A9, neutrophil elastase (ELANE) and matrix metalloprotease 9 (MMP9), all of which are important proteins involved in neutrophil migration (Ryckman et al., 2003) and biological activity, are among predicted atovaquone targets (Figure 1B). Finally, given the potential anti-inflammatory functions of atovaquone, we performed target-based KEGG analysis to identify associations with specific disorders. We found atovaquone targets to be associated with leukemia, hepatitis, colorectal cancer, defects in cytokines and interestingly, neutrophil-signaling associated impairments (Figures 1C,D). These modeling observations predict the potential role of atovaquone in healing inflammation, specifically by targeting neutrophil trafficking.

### Atovaquone treatment promotes resolution of DSS-induced colon injury

**FIGURE 2:**
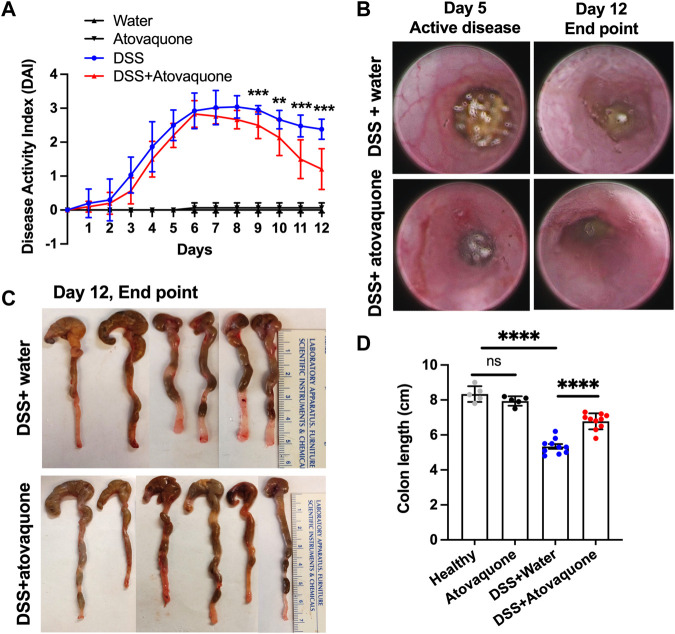
Atovaquone treatment promotes resolution of DSS-induced colon injury. **(A)** Disease Activity Index (DAI) of untreated (blue) or Atovaquone-treated mice (red). N = 2 independent repeats with 5 mice each were analyzed for DSS and DSS + atovaquone conditions. For healthy and atovaquone alone control experiments 5 mice were analyzed. **(B)** Representative high-resolution colon endoscopy images of untreated (top panel) or atovaquone-treated (bottom panel) mice at active disease (day 5) and end point (day 12). At least 4 mice were scoped per condition. **(C,D)** Colon length was measured as an index of inflammation resolution. **(C)** Representative images of dissected colons and **(D)** Quantification of colon length (cm) in untreated (top panel) versus atovaquone-treated (bottom panel) animals. N = 2 independent repeats with 5 mice each were analyzed for DSS and DSS + atovaquone conditions. For healthy and atovaquone alone control experiments 5 mice were analyzed. **p < 0.01, ***p < 0.001, ****p < 0.0001, ns, not significant.

Inflammatory Bowel Disease (IBD) is a risk factor for colon cancer featuring excessive neutrophil-driven inflammation (Lucafo et al., 2021). Since atovaquone was predicted to target colon cancer and neutrophil function, we examined its anti-inflammatory properties in mucosal inflammation resolution. To closely mimic the therapeutic regimen in UC, episodic colitis/colon injury was induced in animal cohorts by administration of 3% DSS (weight/volume) in drinking water. Following 5 days of treatment, DSS was stopped and substituted with either normal water (control) or water containing atovaquone (10 mg/kg). Colitis/injury resolution was then monitored for an additional 7 days by quantifying Disease Activity Index (DAI) and by visual inspection using high-resolution endoscopic imaging. Atovaquone treatment significantly enhanced disease resolution compared to untreated animals (Figure 2A). Improved mucosal healing with atovaquone treatment was further confirmed by endoscopic imaging as shown by representative active disease end-point images (days 5 and 12, Figure 2B) and by quantification of colon lengths, with colonic shortening being a hallmark of colitis (Figures 2C,D). Control atovaquone treatment of healthy mice had no impact on disease activity or colon length (Figures 2A,D). Taken together, these findings demonstrate that atovaquone can be used therapeutically to promote resolution of colonic injury.

### Atovaquone reduces neutrophil burden in inflamed colon

**FIGURE 3:**
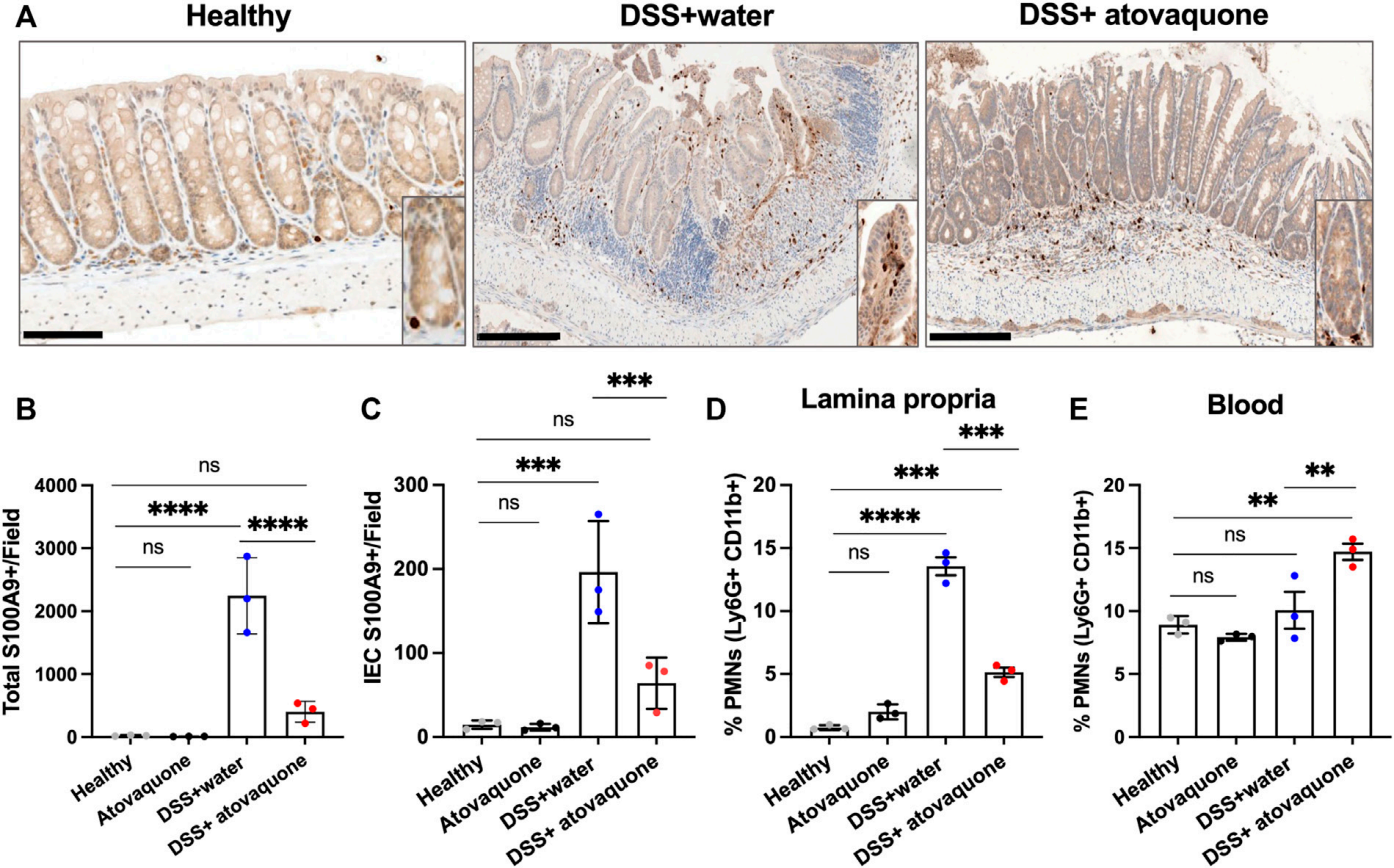
Atovaquone reduces neutrophil burden in inflamed colon. **(A)** Representative immunohistochemistry (IHC) staining for neutrophil marker S100A9 of tissue sections from DSS-treated mice without (top panel) and with Atovaquone treatment (bottom panel). The bar is 100 μm. **(B)** Quantification of total S100A9 positive cells per field and **(C)** Number of intraepithelial S100A9 positive cells (cells that are in direct contact with the epithelial crypts). At least 20 fields per condition (shown as averaged value) from 3 independent experiments were quantified. **(D)** Analyses of neutrophil numbers by flow cytometry within the digested colon lamina propria and **(E)** Peripheral blood of DSS-treated mice with/without atovaquone treatment. Data are shown as percent of Ly6G/CD11b positive neutrophils out of total cells. N = 3 independent repeats. **p < 0.01, ***p < 0.001, ****p < 0.0001, ns, not significant.

Given the predicted impact of atovaquone on neutrophil activity and the role of neutrophils in colon inflammation (Zhou and Liu, 2017), we next asked whether atovaquone improves DSS-induced injury resolution by limiting neutrophil accumulation in inflamed colon. To do so, we quantified neutrophil numbers in DSS-treated colon sections based on IHC staining for the neutrophil marker S100A9. S100A9 has also been identified as one of the atovaquone predicted targets, Figure 1C. Atovaquone treatment of colitic mice significantly reduced the number of S100A9 positive neutrophils in colon tissue as compared to the untreated cohort (Figures 3A,B). Importantly, prior data showed that intraepithelial neutrophil burden is prognostic of response to therapeutics in IBD (Narula et al., 2022). We thus assessed the impact of atovaquone on intraepithelial neutrophil number and found the number of S100A9 positive neutrophils to be significantly reduced (Figure 3A zoom-in insert and Figure 3C). To confirm atovaquone-mediated reduction in colon tissue neutrophils, we performed flow cytometry on digested colon lamina propria (LP). Consistent with IHC analyses, atovaquone treatment led to a significant reduction in Ly6g^+^/CD11b^+^ neutrophils in colon tissue (Figure 3D). The number of circulating Ly6g^+^/CD11b^+^ neutrophils following atovaquone treatment was slightly, but not significantly elevated indicating potential impairment in neutrophil diapedeses (Figure 3E). Atovaquone treatment alone had no impact on the neutrophil burden in healthy mice. Together, our data indicate that atovaquone improves colon injury resolution by suppressing neutrophil migration/tissue accumulation.

### Atovaquone negatively regulates neutrophil migration by downregulating CD11b expression

**FIGURE 4:**
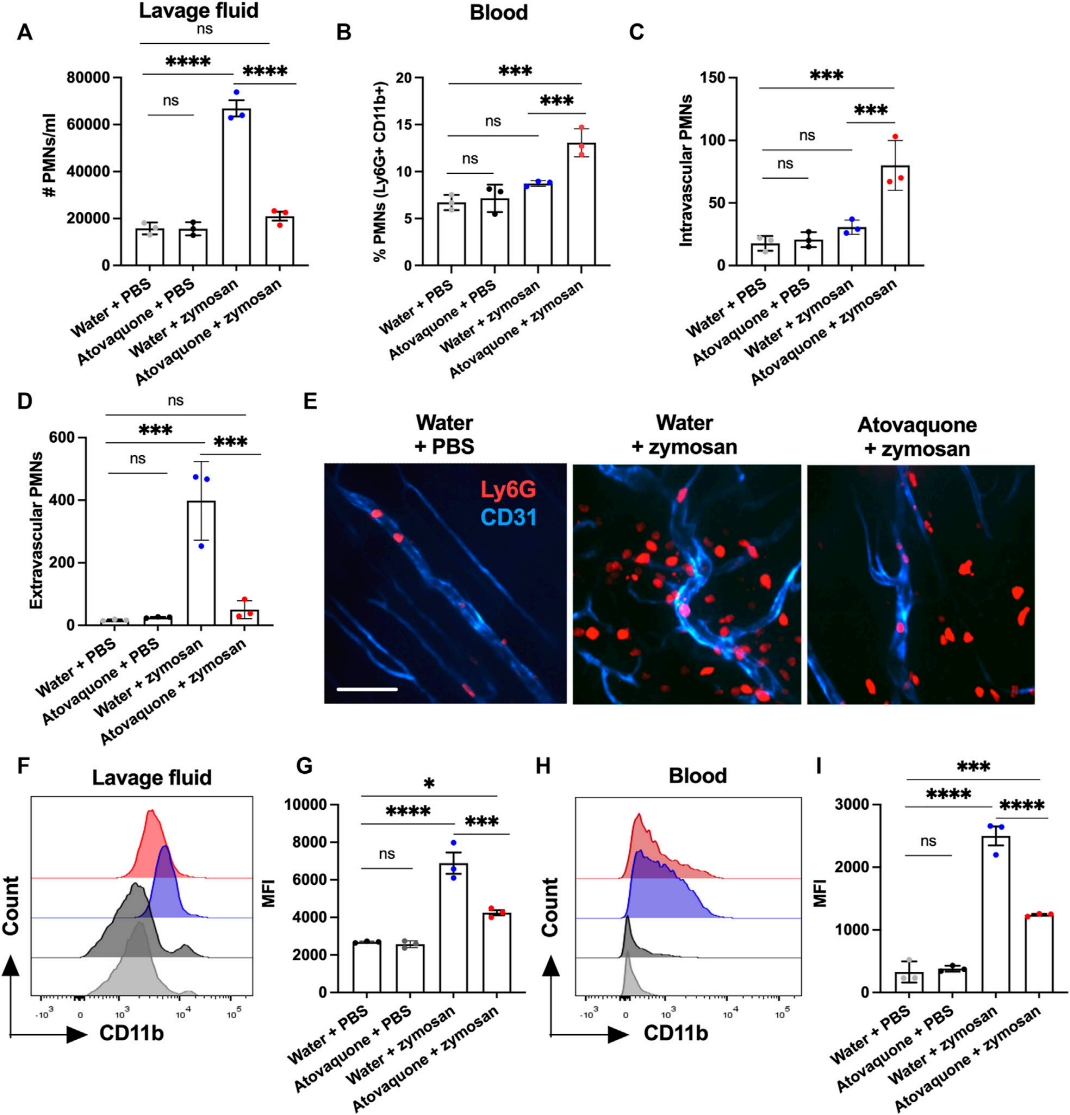
Atovaquone negatively regulates neutrophil migration by downregulating CD11b expression. **(A)** Number of neutrophils in the peritoneal lavage or **(B)** in peripheral blood was quantified by flow cytometry following zymosan-induced peritonitis and with/without Atovaquone treatment (administered in drinking water 2 days prior to zymosan stimulation). N = 3 independent repeats. **(C,D)** Quantification of intravascular/extravascular neutrophils following zymosan-stimulation with/without atovaquone treatment. **(E)** Representative images of mesenteric blood vessels acquired by spinning-disc confocal microscopy. Neutrophils and blood vessels were stained by iv. administration of fluorescently labeled anti-Ly6G (red) and anti-CD31 (blue) antibodies. The bar is 50 μm. At least 15 fields per condition (shown as averaged value) from 3 independent experiments were analyzed. **(F–I)** Mean fluorescence intensity of surface CD11b was quantified by flow cytometry in neutrophils harvested from the peritoneal lavage **(F,G)** and peripheral blood **(H,I)** following zymosan-stimulation with/without atovaquone treatment. N = 3 independent repeats. *p < 0.05, ***p < 0.001, ****p < 0.0001, ns, not significant.

To establish whether atovaquone directly impacts neutrophil migration, zymosan-induced peritonitis neutrophil migration assays were performed. In these experiments, zymosan-A (10 μg) was injected into the peritoneal cavity of mice to induce neutrophil migration, and the number of neutrophils in the peritoneal cavity was quantified by flow cytometry on peritoneal lavage with and without atovaquone pre-treatment (48 h prior to zymosan injection). Atovaquone treatment on its own did not induce neutrophil infiltration, however it significantly reduced neutrophil migration into the peritoneum following zymosan administration (Figure 4A). Consisted with impairment in zymosan-induced neutrophil tissue infiltration, neutrophil numbers in the circulation were elevated with atovaquone treatment (Figure 4B). This was further confirmed through spinning-disc confocal microscopy of mesenteric blood vessels, where we observed significantly reduced numbers of tissue neutrophils and increased number of retained adherent neutrophils within blood vessels in mice treated with atovaquone (Figures 4C–E). CD11b is a key adhesive receptor mediating neutrophil chemotaxis (Szczur et al., 2009; Kumar et al., 2012) and transendothelial migration (Voisin and Nourshargh, 2013). Thus we used flow cytometry to evaluate expression of CD11b in zymosan-elicited neutrophils in the peritoneum lavage and the circulation. With atovaquone treatment, surface CD11b expression (quantified by mean fluorescence intensity, MFI) was significantly reduced compared to the untreated group in both lavage and circulating neutrophils, suggesting that atovaquone attenuates CD11b upregulation and as such limits neutrophil migration (Figures 4F–I).

**FIGURE 5:**
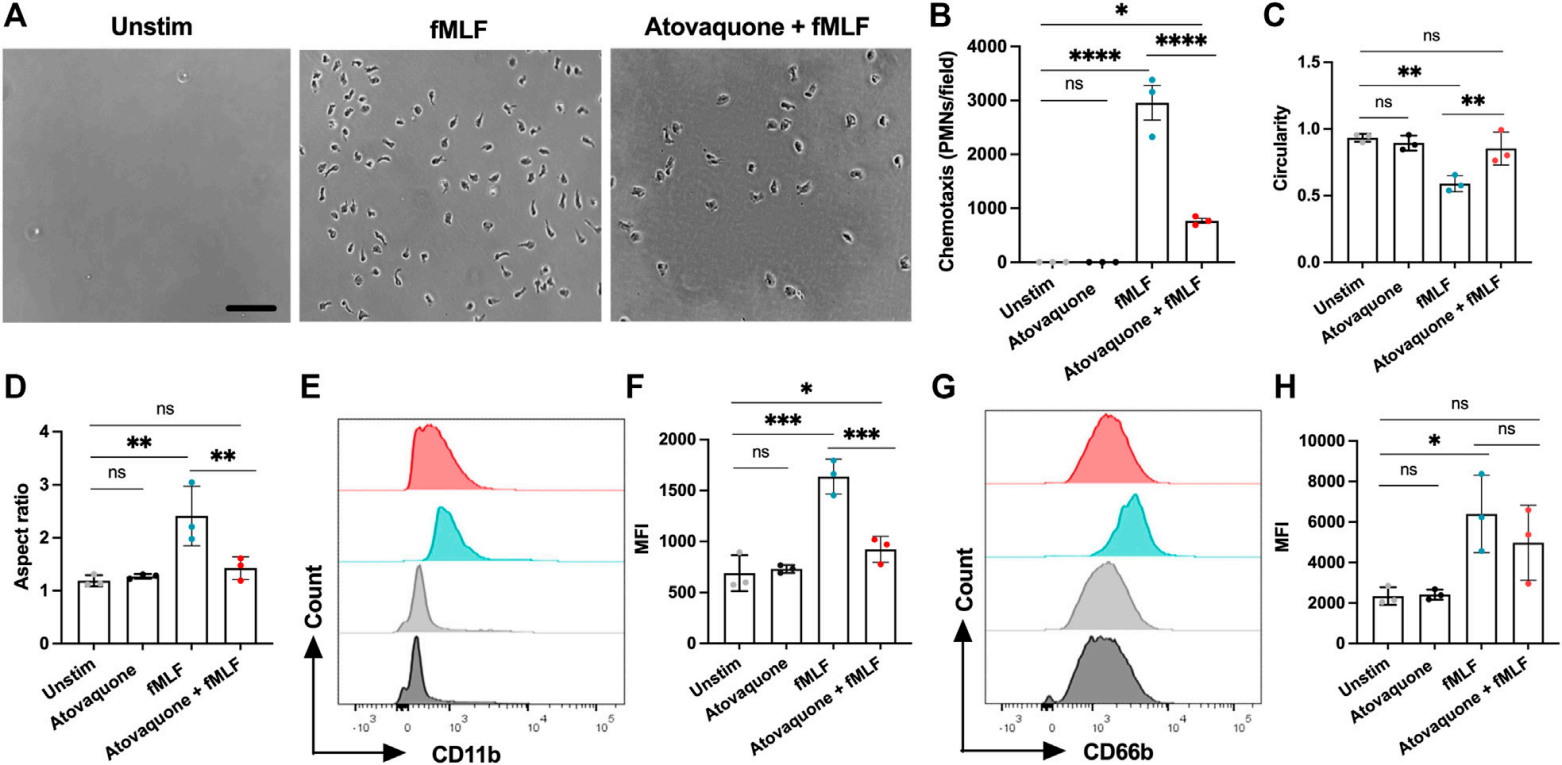
Atovaquone negatively regulates human neutrophil chemotaxis by downregulating CD11b expression. **(A–D)** Freshly isolated human neutrophils were induced to migrate across permeable supports by introduction of the fMLF gradient. **(A)** Representative bright field images (the bar is 50 μm) and **(B)** Quantification of neutrophils following chemotaxis in the bottom chamber with/without pre-treatment with atovaquone. At least 7 fields per condition were quantified from 3 independent experiments that were performed in duplicates. Data are shown as an averaged value of all fields in both duplicates. **(C,D)** Analyses of neutrophil polarization [indexed by **(C)** circularity and **(D)** length to width ratio] following chemotaxis with/with pre-treatment with atovaquone. At least 7 fields per condition were quantified from N = 3 independent repeats in duplicates. **(E–H)** Human neutrophils, untreated or pre-treated with atovaquone were stimulated with fMLF (200 nM, 30 min). **(E,F)** Representative flow diagram and quantification of mean fluorescence intensity (MFI) of surface CD11b. **(G,H)** Representative flow diagram and MFI quantification of surface CD66b. N = 3 independent repeats using 3 different donors. *p < 0.05, **p < 0.01, ***p < 0.001, ****p < 0.0001, ns, not significant.

Since atovaquone is an FDA-approved therapeutic for human use, we next used a transwell setup to evaluate its impact on the chemotaxis of human peripheral blood neutrophils. In these experiments, freshly isolated human neutrophils were pre-treated with atovaquone (1 μM, 20 min), introduced to the upper chambers of transwells and induced to migrate across permeable supports towards an *N*-Formylmethionyl-leucyl-phenylalanine (fMLF) gradient (200 µM, added to the bottom chamber). Consistent with murine studies, we found that while atovaquone treatment on its own had no significant impact on neutrophil migration, atovaquone treatment significantly reduced neutrophil migration towards fMLF gradient as compared to untreated neutrophils (Figures 5A,B). Furthermore, we observed that atovaquone treatment impacted neutrophil polarization and spreading, typical of activated neutrophils. Following migration, atovaquone-treated neutrophils were significantly more round (increased circularity index) and less polarized (decreased aspect ratio) compared to migrated untreated cells (Figures 5C,D). In control experiments, we established that atovaquone treatment on its own (without migration) had no impact on neutrophil morphology and polarization (Figures 5C,D).

Finally, given our observation of decreased CD11b expression by atovaquone treatment in murine neutrophils, and the previously demonstrated role for CD11b in neutrophil polarization and migration, we asked if atovaquone similarly prevented inflammatory cue driven CD11b upregulation in human neutrophils. Surface expression of the active form of CD11b on human neutrophils (antibody used specifically recognizes a high affinity conformation state of the CD11b) was examined by flow cytometry with and without atovaquone pre-treatment (1 μM, 20 min) followed by stimulation with fMLF (200 μM, 30 min). Atovaquone pre-treatment significantly attenuated fMLF-induced CD11b upregulation (Figures 5E,F), indicating reduced activation. Expression of CD66b, which is a marker of neutrophil activation *via* degranulation (Lacy, 2006), trended downward but did not reach statistical significance (Figures 5G,H). Consistent with morphological analyses, atovaquone treatment alone had no significant impact on human neutrophil CD11b expression, indicating no direct activation by the drug. Together these findings suggest that atovaquone impacts migratory activation rather than neutrophil degranulation.

## Discussion

Dysfunctional immune responses, featuring aberrant T cell activation and neutrophil activity are the major drivers of IBD, therefore targeting the immune system has been of ongoing therapeutic interest. Several biologics have been recently developed and implemented in clinic to target immune cell-driven inflammation. This includes immunomodulatory inhibitors of TNFα and IL12/23, as well as inhibitors of α4β7 integrin, specifically targeting T cell recruitment.

During disease, neutrophils are recruited to the inflamed mucosa and the luminal space in response to inflammatory cues *via* several tightly regulated sequential steps which include crossing of the endothelial and the epithelial barriers (Voisin and Nourshargh, 2013; Sumagin et al., 2014; Sullivan et al., 2018). Importantly, most of the neutrophil effector functions as well as their pathological effects occur after they leave the circulation and enter the surrounding tissue. Thus, targeting neutrophil trafficking is of therapeutic relevance. Although the role of neutrophils in IBD pathology is well established (Lee et al., 2018), no drugs to date have been developed or studied in IBD to target neutrophil recruitment and/or activity.

Antimalarial drugs have been used safely for decades to treat inflammatory diseases, such as systemic lupus erythematosus and rheumatoid arthritis. Atovaquone, an FDA-approved antimalarial drug, has shown tumor-suppressive and anti-viral properties *in vitro* (Fiorillo et al., 2016; Carter-Timofte et al., 2021). Nevertheless, its role in gut inflammation has not been studied before. In this study, we evaluated for the first time the role of atovaquone in gut inflammation using a combination of predictive modeling, a pre-clinical DSS-induced colitis model and mechanistic studies using human primary immune cells. Our results demonstrate that atovaquone promotes resolution of DSS-induced murine colitis *via* novel mechanism of targeting neutrophil activity, indicating its potential use for IBD therapy.

Unbiased predictive modeling approach revealed unique targets for atovaquone. Among the top targets we found molecules known to be induced during inflammatory diseases as well as signaling pathways associated with leukocyte trafficking across endothelial cells, and specific neutrophil activation. We found that atovaquone directly inhibits neutrophil migration and accumulation in inflamed colonic mucosa in the DSS-induced colitis model, demonstrating that it aids in colitis resolution by reducing neutrophil-associated tissue damage. We confirmed the atovaquone direct impact on neutrophil migration using zymosan-induced peritonitis mode, where we observed a potent atovaquone-mediated suppression in neutrophil accumulation in the peritoneal cavity in response to zymosan stimulation. Given the rapid dynamics of this model (4 h stimulation), the decrease in neutrophil numbers likely resulted from suppressed neutrophil ability to transmigrate and cross the endothelial barrier. Indeed, flow cytometry and confocal microscopy analyses confirmed elevated numbers of neutrophils within blood vessels and significantly reduced tissue infiltration following atovaquone treatment. These observations mechanistically support our data from the DSS-induced colitis model, where atovaquone treatment led to improved disease resolution, and based on histological and flow cytometry analyses, significantly reduced neutrophil infiltration of the colonic mucosa. We have previously shown in the same model that neutrophil activity in the inflamed colonic mucosa was imperative to colon injury resolution due to neutrophil mediated inhibition of DNA double strand break repair. Reducing neutrophil tissue accumulation *via* an antibody-based depletion approach improved wound healing (Butin-Israeli et al., 2019), supporting our current data.

To make our observations more clinically relevant, we also used isolated primary human neutrophils to demonstrate that atovaquone in approved dosing concentrations indeed directly suppressed neutrophil chemotaxis. Intriguingly, we also noted impaired neutrophil polarization and spreading in the presence of atovaquone, indicating a decreased degree of activation and/or impaired adhesive interactions. CD11b is a neutrophil adhesive ligand for endothelial ICAM-1, playing a critical role in neutrophil adhesion and transendothelial migration (Filippi, 2019). CD11b has been also shown to mediate activation and polarization of migrating neutrophils. As such, CD11b deficient neutrophils exhibit impairment in lamellipodium and uropod formation, which are necessary for the generation of cell polarity, leading to defective migration (Szczur et al., 2009; Kumar et al., 2012; Voisin and Nourshargh, 2013). Based on these observations we hypothesized that atovaquone could be negatively impacting neutrophil migration by downregulating CD11b. Consistent with this idea, analyses of murine peritoneal neutrophils revealed significant downregulation of CD11b expression with atovaquone treatment. Furthermore, atovaquone pre-treatment of human neutrophils prevented an fMLF-induced upregulation of the active form of CD11b, confirming that by yet unknown mechanism, atovaquone prevents upregulation of CD11b to attenuate neutrophil migration. CD11b is stored in secretory vesicles and its release to the cell surface is regulated by several molecular pathways, including actin cytoskeleton rearrangements through RhoA (Anderson et al., 2000) and Cdc42 (Szczur et al., 2009; Kumar et al., 2012), ERK 1/2 (Jozsef et al., 2002) and S100A9 (Scott et al., 2020) signaling pathways. In future work, we plan to follow up on this exciting observation and to decipher which specific pathway/molecules are targeted by atovaquone. Many neutrophil-associated pathologies, including vascular dysfunction or epithelial injury involve degranulation and release of damaging enzymes, such as elastase, myeloperoxidase and matrix metalloproteinases (Sheshachalam et al., 2014). Interestingly, neutrophil degranulation was not significantly impacted by atovaquone, as indicated by surface CD66b expression (Lacy, 2006), demonstrating specificity to neutrophil migratory activation.

Taken together, our data identified a novel role for atovaquone in attenuating neutrophil migration by targeting CD11b. Our data established the anti-inflammatory/pro-resolution effect of atovaquone in mucosal inflammation/injury, supporting further study into potentially repurposing this FDA-approved drug as a therapy for IBD.

## Data Availability

The original contributions presented in the study are included in the article/Supplementary Material, further inquiries can be directed to the corresponding authors.
